# Production of Chitin from *Penaeus vannamei* By-Products to Pilot Plant Scale Using a Combination of Enzymatic and Chemical Processes and Subsequent Optimization of the Chemical Production of Chitosan by Response Surface Methodology

**DOI:** 10.3390/md15060180

**Published:** 2017-06-16

**Authors:** José A. Vázquez, Patrícia Ramos, Jesús Mirón, Jesus Valcarcel, Carmen G. Sotelo, Ricardo I. Pérez-Martín

**Affiliations:** 1 Grupo de Reciclado y Valorización de Materiales Residuales (REVAL), Instituto de Investigacións Mariñas (IIM-CSIC) r/Eduardo Cabello, 6, Vigo 36208, Galicia, Spain; suso@iim.csic.es (J.M.); jvalcarcel@iim.csic.es (J.V.); 2 Grupo de Bioquímica de Alimentos, Instituto de Investigacións Mariñas (IIM-CSIC) r/Eduardo Cabello, 6, Vigo 36208, Galicia, Spain; ariza@iim.csic.es (P.R.); carmen@iim.csic.es (C.G.S.); ricardo@iim.csic.es (R.I.P.-M.)

**Keywords:** chitin, chitosan, *Penaeus vannamei* shells, response surface methodology, by-products valorization

## Abstract

The waste generated from shrimp processing contains valuable materials such as protein, carotenoids, and chitin. The present study describes a process at pilot plant scale to recover chitin from the cephalothorax of *Penaeus vannamei* using mild conditions. The application of a sequential enzymatic–acid–alkaline treatment yields 30% chitin of comparable purity to commercial sources. Effluents from the process are rich in protein and astaxanthin, and represent inputs for further by-product recovery. As a last step, chitin is deacetylated to produce chitosan; the optimal conditions are established by applying a response surface methodology (RSM). Under these conditions, deacetylation reaches 92% as determined by Proton Nuclear Magnetic Resonance (^1^H-NMR), and the molecular weight (Mw) of chitosan is estimated at 82 KDa by gel permeation chromatography (GPC). Chitin and chitosan microstructures are characterized by Scanning Electron Microscopy (SEM).

## 1. Introduction

Prawns and shrimp are the second fish product marketed at world level, and constitute 8% of the total value of internationally traded fish products. In aquaculture, *Penaeus vannamei* is the top cultured species. Because of its great commercial value, the food industry processes a significant amount of these crustaceans, in many cases involving the removal of the exoskeleton of the tail and the cephalothorax. As a result, about 40–45% of the whole animal is considered by-products. This low yield combined with the commercial importance of this species results in high amounts of waste generated by its industrial processing. Therefore, alternatives for the treatment of these by-products are necessary, ideally scaled at pilot plant level to assess their viability.

The by-products generated contain compounds of interest, such as protein, pigments, and chitin. In particular, the exoskeleton represents the main source of chitin and chitosan [1]. The former is the second most abundant natural biopolymer after cellulose, and is one of the major sources of surface pollution in coastal areas. The biosynthesis of chitin involves the linkage of *N*-acetylglucosamine units by β-(1→4) glycosidic bonds to form a long-chain polymer. The arrangement of chitin chains in crustacean shells is antiparallel (α-chitin) with strong intra- and inter-molecular forces. This structure makes chitin insoluble in most solvents, but can be increased by partial *N*-deacetylation via an alkaline treatment to yield chitosan.

Chitin and its cationic derivative chitosan display functional and physicochemical properties that offer a wide range of industrial applications, and potentially solve numerous problems in the environmental, agricultural, cosmetic, and biomedical fields [2,3,4,5]. In particular, the food applications of chitin and chitosan are numerous, and include food preservation against microbial deterioration due to its antimicrobial activity; the formation of biodegradable films; and the clarification and deacidification of fruit juices, etc. [6]. In fish, chitosan coatings display preservative properties in herring and cod, reducing moisture loss, lipid oxidation, and microbial growth [7,8].

Protocols for the purification of chitin from the endoskeletons and exoskeletons of crabs, squid, shrimp, etc., usually include steps of deproteinization, demineralization, and, in some cases, bleaching [9,10]. Secondary products such as extract hydrolysates and carotenoids can also be recovered from the residual effluents generated [11,12,13]. The subsequent reaction of deacetylation to transform chitin into chitosan is commonly mediated by alkaline treatments at temperatures higher than 100 °C [14,15,16,17]. Conventional methods for chitin and chitosan production are energy intensive, and require hazardous chemical compounds [18,19]. Safer and more environmentally friendly alternatives, based on the combination of microbial, enzymatic, and chemical strategies, have recently been reported [20,21,22,23]; but to date, the only studies about chitin purification at pilot plant or industrial scale are patents. Concise reports on the optimization of chitosan production from *P. vannamei* materials are practically nonexistent [24]. In this context, response surface methodology (RSM) is one of the most effective mathematical tools in order to evaluate the joint effect of several independent variables, and to maximize or minimize the production of the dependent variable object of study [25,26,27].

This work aims to provide a detailed report on the production of chitin from industrial by-products of *P. vannamei* at pilot plant scale, and the optimization of chitosan transformation from the chitin previously extracted. A combination of enzymatic–acid–alkaline treatments is developed for the purification of chitin, taking advantage of recent progress in deproteinization strategies with proteases. Short processing times, low temperature, and reduced alkali and acid concentrations complete the sequence to achieve a more sustainable process. In this same line, optimal conditions for chitosan production are established, based on response surface methodology. In all cases, the composition and physicochemical characteristics of the substrates and products are evaluated to ascertain their purity and quality.

## 2. Results and Discussion

### 2.1. Pilot Plan Production of Chitin

**Figure 1:**
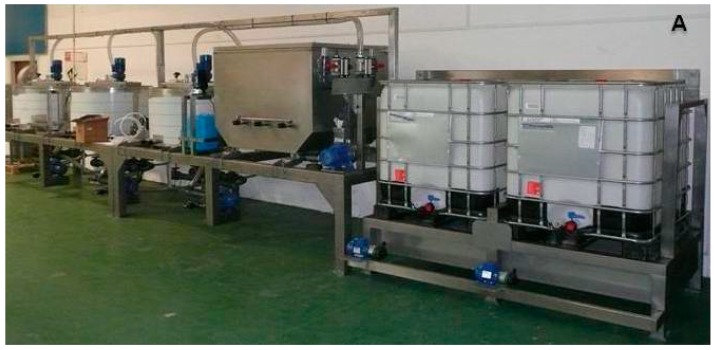

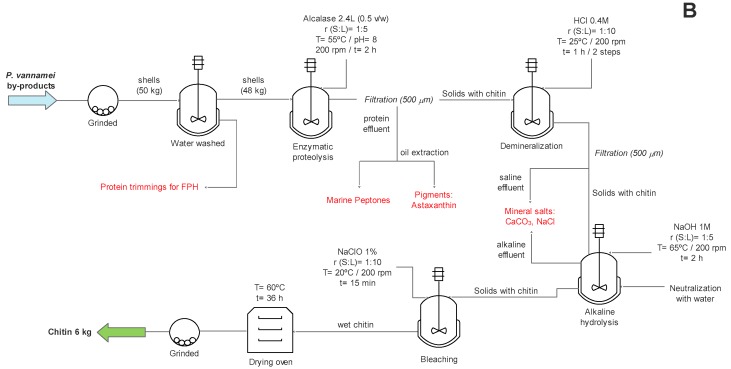
Photograph of the pilot plan developed for the present study (**A**); Flowchart of chitin production at pilot plan scale combining enzymatic and chemical steps (**B**).

The moisture, ash, protein, and lipid contents of shrimp by-products were 76%, 4.76%, 55% and 1.6% respectively (Table 1). The compositional values are similar to the samples described in previous reports [28,29]. The process applied in the present work for the industrial production of chitin (Figure 1) is an improved modification of the alkaline–enzymatic methods reported by other authors [28,30,31,32]. These improvements are based on the reduction of the concentrations of alkaline (1 M NaOH) and acid reagents (0.4 M HCl), time (6 h for the whole process), and processing temperature (less than 65 °C), when compared to the more extreme conditions described in the cited works. The chitin thus obtained presents similar purity levels in terms of the ash content, the degree of acetylation, and the infrared fingerprint, which demonstrates that milder conditions are sufficient to obtain high quality chitin (Table 1).

The degree of *N*-acetylation can be derived from the carbon/nitrogen ratio (*C/N*), which is a fundamental parameter for discriminating between chitin and chitosan. Theoretically, that ratio is 6.9 for fully acetylated chitin, and 5.1 for fully deacetylated chitosan. The degree of acetylation (*DA*) was calculated by the following equation [33]:

(1) $$DA\left(\%\right)=\frac{\left(C/N-5.14\right)\times 100}{1.72}.$$

It resulted in a *DA* of 96%, and *C/N* = 6.69 (Table 1) for the dry solids obtained at the end of the process line, which confirms its identity as chitin.

**Figure 2:**
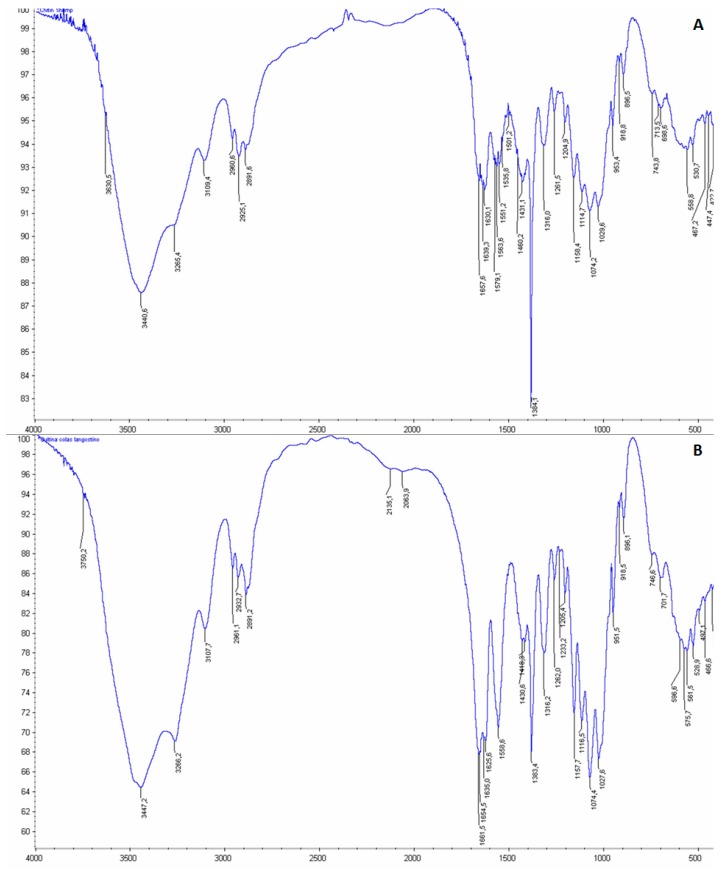
Infrared (IR) spectrum of chitin pattern provided by Sigma-Aldrich (**A**); IR spectrum of chitin from *P. vannamei* by-products (**B**).

Figure 2 shows the comparison of the infrared spectra (IR) of the final product obtained from commercial chitin (A), and *P. vannamei* by-products (B). The identification of the characteristic chitin bands OH group at 3440–3447 cm^−1^; NH group at 3265–3266 cm^−1^; the CH band at 2927–2933 cm^−1^; and the amide I band at 1630 cm^−1^ [5,34], as well as the similar pattern, confirms chitin presence in the final product, in agreement with the DA results. In addition, the ash percentage was low (1.44%), which reveals an almost complete demineralization of the sample under the conditions used in the process, and is consistent with the IR spectrum, free from interfering bands.

**Figure 3:**
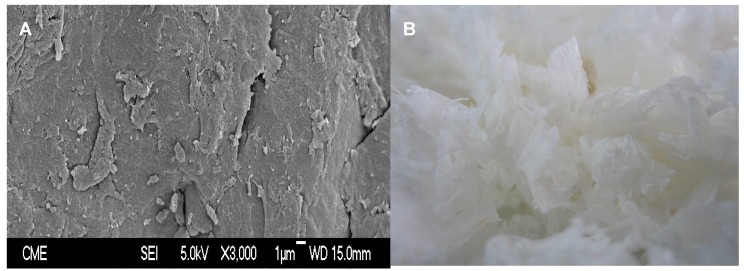
Image of SEM of chitin (**A**) and normal pictures of white flakes of purified chitin (**B**).

The scanning electron microscope (SEM) image of chitin displays the conventional surface with irregular shapes and heterogeneous size, observing asymmetric particles or microfibrils (Figure 3A) [35]. The linear sequence of *N*-acetylglucosamine monomers linked by covalent β-(1→4) glycosidic bonds is considered the primary structure of chitin. These chains are joined together by multiple hydrogen bonds in three-dimensional microfibrils, which constitutes their secondary structure. Moreover, microfibrils bind to proteins, and create the tertiary structure of chitin. The percentage of crystalline material in chitin (crystallinity index, ICr), calculated according to Segal et al. [36], is 88.1 ± 3.7%. The white flakes of chitin produced at the pilot plant are depicted in Figure 3B. The final yield of chitin recovery reaches 30%.

The results from the elemental analysis—including heavy metals—are also summarized in Table 1. The low values obtained agree with the low ash content in the final chitin samples. The Pb, Hg, and Cd levels are consistent with data previously reported [37], and comply with the values established by European Union legislation [38].

Leaving aside unclear patent references, this is the first approach to the scaled-up production of chitin from waste generated in the industrial processing of *P. vannamei* (Figure 1A). Additionally, the effluents generated in the process can either represent inputs for other valorization processes, or be safely discarded (Figure 1B). The first effluent and secondary by-product consists of the protein trimmings obtained in the initial water washings, which could be an interesting substrate, after filtration, for the production of fish protein hydrolysates (FPH). Its high protein content (more than 60%) and the presence of essential amino acids make this waste a candidate to be enzymatically processed to be applied as a high quality nutrient in aquaculture feed [39,40,41].

Subsequent to the enzyme proteolysis of shells and hydrolysate filtration, two fractions are recovered: (a) solids containing chitin, mineral salts, pigments, and remaining protein, later used as a substrate for chitosan production; (b) an effluent rich in soluble proteins and pigments, which can be processed with vegetal oil for the recovery of pigments—mainly astaxanthin—in the oil fraction [42,43]. The later remaining aqueous fraction contains a remarkable amount of protein (6.95 ± 0.22 g/L), which is possible to separate after centrifugation. Recently, the extraction of pigments from the wastewaters of the industrial processing of *P. vannamei* shells has been extensively reported [44]. Such approaches are mainly based on vegetal oil extraction at low–medium temperatures (40–70 °C), sometimes combined with previous concentration steps by ultrafiltration membranes and hydrolysis assisted by commercial proteases. Regarding the protein effluent, it may be an excellent organic nitrogen source (marine peptones) for the culture of several bacteria (e.g., marine probiotics, lactic acid bacteria, etc.). This was confirmed in a recent study using effluents obtained from the enzymatic β-chitin production from the pens of *Illex argentinus* squid [12].

The effluents from acid demineralization and alkaline hydrolysis are mixed to achieve neutral pH, reducing thereby the difficulty and the cost of managing strong acid and alkaline solutions. Furthermore, this effluent is a good source of calcium (in carbonate and phosphate forms) and sodium (as chloride), which are of increasing interest as food supplements of marine origin [45,46]. Finally, the bleaching solution, with a slight orange coloration, can be discharged (or employed for washing the pilot plant) without previous depuration.

### 2.2. Optimization of Chitosan Production

The habitual procedure for chitin deacetylation is mediated by the alkaline hydrolysis of the acetyl groups at high temperature. Although it is not a particularly environmentally friendly process, enzymatic alternatives using deacetylases are not a realistic option nowadays, since no commercial deacetylases are available. Recent studies of fermentative deacetylation using fungus, such as *Mucor rouxii,* have shown promising results, but crystalline chitin must be necessarily pretreated prior to enzyme hydrolysis to improve the accessibility of the enzyme to the acetyl groups [47]. The applications of the mentioned deacetylases, directly or by fungus fermentation, are not yet used at an industrial scale.

**Figure 4:**
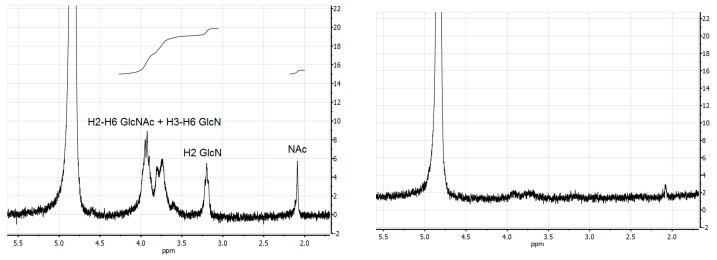
^1^H-NMR spectra for chitosan with high *DD* obtained in the experimental condition *t* = 24 h/NaOH = 50% (left) and low *DD* obtained in the experimental condition *t* = 20.6 h/NaOH = 35.9% (right). GlcNAc: *N*-acetylglucosamine, GlcN: glucosamine, NAc: *N*-acetyl.

In the present work, a two-factor rotatable design has been performed to study the effect of two independent variables (time and alkali concentration) in the maximization of chitosan production from chitin of *P. vannamei* waste (Table 2). The main response evaluated is the degree of deacetylation (*DD*) quantified by Proton Nuclear Magnetic Resonance (^1^H -NMR). In this context, ^1^H-NMR spectroscopy is the technique employed to determine the structural composition and purity of the polysaccharide (chitosan), based on the intensities of the ^1^H absorption peaks of the corresponding chitosan chemical shifts (in ppm), with the residual HOD solvent signal as reference. Two examples from the factorial design are depicted in Figure 4. For a highly deacetylated sample (left) obtained after reaction with 50% NaOH for 24 h, the ^1^H-NMR spectrum shows the common peaks of *N*-acetyl at 2.09 ppm and H2 of glucosamine (GlcN) at 3.20 ppm. The unresolved signals for protons H2-H6 of *N*-acetylglucosamine (GlcNAc) and H3-H6 of GlcN appear in the region 3.5–4.0 ppm. The signal of AcOH, tipically seen at 2.11 ppm is absent, indicating very little hydrolysis in the dissolved samples. Similar profiles were observed for chitosan produced under other experimental conditions, such as 12.5 h/50% NaOH (spectra not shown). In these cases, DD values are determined from the relative integrals of acetyl (*N*-acetyl and AcOH) and combined H2-H6 protons of, GlcN and GlcNAc [48,49].

In the spectrum on the right, obtained at 20.6 h/35.9% NaOH, the picture is quite different. The typical signals for chitosan described above show very low intensities, corresponding to also low quantities of chitosan in solution. This indicates that only minute amounts of chitin were converted to chitosan, probably because the conditions applied were too mild to render the material soluble, remaining for the most part as chitin. In this context, the weakness of the signals do not allow to calculate values for *DD*, so in these cases the equation proposed by Ottøy et al. [50] is used instead.

The *DD* values obtained by both methods are fitted by the polynomial model (2), followed by a statistical analysis using response surface methodology (Table 2).

The outcomes of the multivariate analysis indicate that the statistical signification of coefficients, evaluated by a Student *t*-test (α = 0.05), is only relevant for the quadratic term of the NaOH and the joint effect of time and alkali. The value of the coefficient of determination adjusted is remarkable ($R_{adj}^{2}$ = 0.817), revealing a good correlation between experimental and predicted data, and indicating that the variability of the *DD* is explained satisfactorily by the second order equation (Table 2). The four F-Fisher tests evaluated are significant, confirming the robustness of the polynomial equation (2).

(2) $$DD=87.37+12.69\;t+4.54\;NaOH+20.25\;t\;NaOH-14.64\;t^{2}-31.74\;NaOH^{2}$$

**Figure 5:**
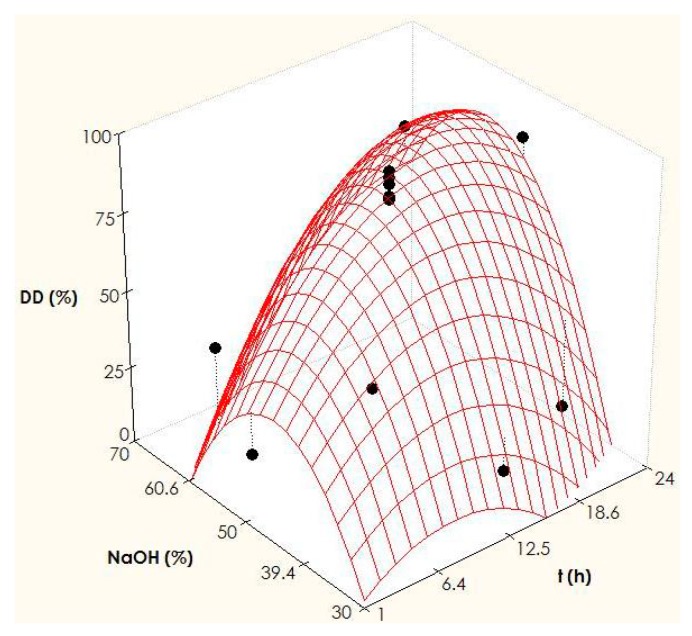
Experimental data (points) and theoretical response surfaces describing the joint effects of alkali and processing time on chitosan production from *P. vannamei* chitin.

Figure 5 displays the experimental data of the *DD*, and the predicted surfaces generated by the model. Based on this equation, the optimal conditions calculated by numerical derivation [51,52] are NaOH = 53.8% and *t* = 17.5 h. The degree of deacetylation calculated in these values is 92%.

The molecular weights (Mw) of various samples from the previous factorial design were also determined, by gel permeation chromatography (GPC). The values of the Mws are different depending on the experimental conditions applied, ranging from 58 to 82 kDa for the more deacetylated samples to 91–93 kDa for the acetylated ones (basically non-deacetylated chitin).

**Figure 6:**
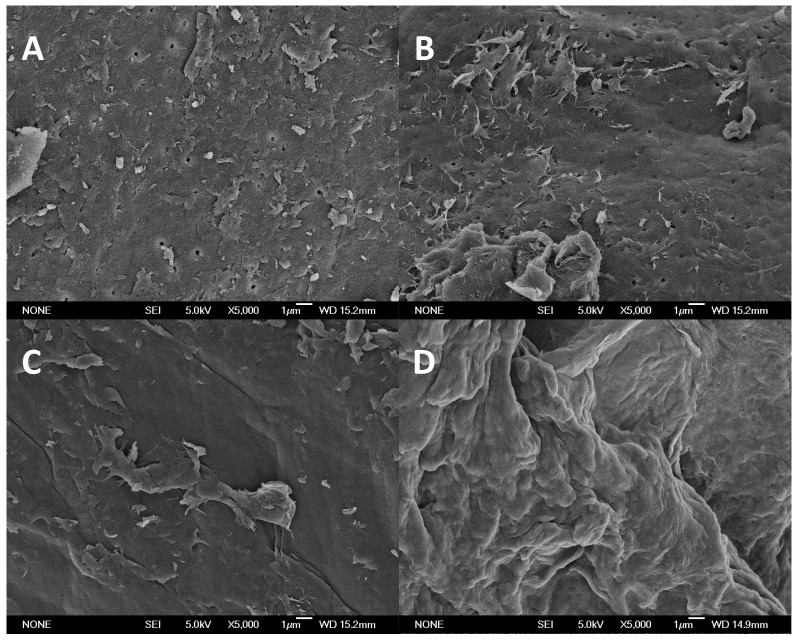
Images of SEM for chitin (**A**,**B**) and chitosan (**C**,**D**) samples obtained at different experimental conditions. (**A**) 12.5 h/NaOH 30%; (**B**) 20.6 h/NaOH 35.9%; (**C**) 12.5 h/NaOH 50% and (**D**) 24 h/NaOH 50%.

Higher Mw chitosan (150 kDa) has been obtained working at higher temperatures and lower NaOH concentrations using the shrimp wastes of non-defined species [52]. A structural analysis by SEM was also performed in four samples (Figure 6). The top images (A and B) also showed irregular patterns, with the presence of microfibrils indicating a perfect crystal structure as a result of a regular packing of the chains corresponding to chitin. In fact, when comparing these images with the obtained crystallinity index (86% and 85%) and the values of the *DD* (20% and 19%), it is evident that they are chitin. In the bottom images (C and D), less crystallinity was observed, in agreement with the data of the *DD* (84% and 85%, respectively).

## 3. Experimental Section

### 3.1. Raw Materials and Extraction of Chitin

The shrimp (*Penaeus vannamei*) by-products, mainly exoskeleton of cephalothorax, were kindly provided by Pescanova S.A. (Vigo, Spain) and stored at −20 °C until use. The purification of the chitin was performed in a pilot plant built for this purpose by Grupo Josmar S.L. (Pontevedra, Spain). This plant consists of three conical stainless steel reactors (each one 500 L), fully equipped with controllers of pH, temperature, and agitation as well as auxiliary equipment (transfer pumps, membrane filters, spray-dryer, etc.) designed for the main treatment of crustacean by-products to produce chitin (Figure 1, top): (a) enzymatic proteolysis (by Alcalase); (b) acid demineralization (by HCl); and (c) alkaline hydrolysis (using NaOH) and chemical bleaching (by NaClO). The commercial chitin from shrimp shells (reference C7170) was purchased from Sigma-Aldrich SA (St. Louis, MO, USA).

The chitin production line was performed (in duplicate) using batches of 50 kg of shrimp by-products (Figure 1, bottom). Initially, these materials were ground (0.5–1.5 cm) and washed to eliminate protein trimmings from the rest of the crustacean’s body. Subsequently, the shrimp exoskeleton was hydrolysed by a commercial protease, Alcalase 2.4L (Novozymes S.A.), at an enzyme concentration of 0.5 (*v*/*w* of substrate) in a solid:liquid ratio of 1:5 (*w*/*v*) with distilled water at 55 °C and pH = 8. A constant pH was maintained for 2 h by the addition of 1M NaOH as required. At the end of the enzyme proteolysis, the solids were separated from the protein effluent by filtration (500 µm), and passed on to the next step: acid treatment with 0.4 M HCl using solid:liquid ratio of 1:10 (*w*/*v*) for 1 h under agitation (200 rpm) at room temperature. This second stainless steel reactor is internally covered with an epoxy material to prevent a strong acid attack. After the acid treatment, the effluent was filtered and the solid was washed until neutral pH. The acid treatment was performed twice. The last stage was an alkaline treatment with NaOH 1M using a ratio 1:5 (*w*/*v*) for 2 h at 65 °C, then the solid was washed to neutrality and treated with 1% sodium hypochlorite (ratio 1:10 *w*/*v*) for 15 min at room temperature. Finally, the solution was filtered and the resulting solids (chitin) were dried (by drying oven) at 60 °C for 24 h and milled.

### 3.2. Compositional Characterization of P. vannamei By-Products and Produced Chitin

Different methods were applied to characterize both the shrimp cephalotorax and the purified chitin: the water content was determined by drying at 105 °C until constant weight; the total nitrogen was analyzed according to the Kjeldahl procedure [53]; the ash content was assessed according to the AOAC protocol [53]; and all of the lipids were extracted by the Bligh and Dyer method [54].

In chitin, the presence of ions was quantified by inductively coupled plasma mass spectrometry (ICP-MS), and the heavy metal content by atomic absorption spectroscopy (AAS). The IR measurements were performed at room temperature on a Thermo Nicolet 6700 FTIR infrared spectrometer. The samples were prepared in a KBr pellet at a ratio sample/KBr of 1:10, and measured in transmission mode in the 400 to 4000 cm^−1^ range using a DTGS type detector (deuterated triglycerin sulfate) and a divider of KBr beam. The C/N ratio was determined by combustion at 1200 °C in a LECO CN-2000 analyzer.

### 3.3. Experimental Design for the Production of Chitosan and Statistical Analysis

A second order rotatable design with quintuple replication in the centre of the experimental domain was performed [55], in order to maximize the deacetylation of the chitin to produce chitosan (Table 1). The combined effect of NaOH (in %) and the time of hydrolysis (*t*, in h) on chitosan production was studied, using as dependent variables (responses) the degree of deacetylation (*DD*) and the solubility (*S*) in acetic acid of the resulting solids. The experimental conditions varied between 1 and 24 h for *t* and 30–70% for NaOH; the solid:liquid ratio (1:20) and temperature (90 °C) were kept constant. The experimental units were 300 mL Erlenmeyer flasks with 5 g of chitin and 100 mL of NaOH solution, each unit fully sampled at the established time intervals. The samples were centrifuged, the supernatant stored at −20 °C, and the solid dried at 60 °C for 24 h and stored in sealed bags for further characterization.

The experimental results of the factorial designs were modelled by second-order polynomial equations, as:

(3) $$Y=b_{0}+\sum_{i=1}^{n}b_{i}X_{i}+\sum_{\begin{array}{l} i=1\\ j>i \end{array}}^{n-1}\sum_{j=2}^{n}b_{ij}X_{i}X_{j}+\sum_{i=1}^{n}b_{ii}X_{i}^{2}$$

where *Y* represents the response to be modelled; *b*_0_ is the constant coefficient, *b_i_* is the coefficient of linear effect; *b_ij_* is the coefficient of interaction effect; *b_ii_* the coefficients of squared effect; *n* is the number of variables; and *X_i_* and *X_j_* define the independent variables.

The goodness-of-fit was established as the adjusted determination coefficient ($R_{adj}^{2}$), the statistical significance of the coefficients was verified by means of the Student *t*-test (α = 0.05), and the model consistency by the Fisher *F* test (α = 0.05), using the following mean squares ratios:
**Mean Squares Ratios****the Model Is Acceptable When***F*1 = Model/Total error
$F1\geq F_{den}^{num}$*F*2 = (Model + Lack of fitting)/Model
$F2\leq F_{den}^{num}$*F*3 = Total error/Experimental error
$F3\leq F_{den}^{num}$*F*4 = Lack of fitting/Experimental error
$F4\leq F_{den}^{num}$

$F_{den}^{num}$ are the theoretical values to α = 0.05 with the corresponding degrees of freedom for the numerator (num) and denominator (den). All of the fitting procedures, coefficient estimates, and statistical calculations were performed on a Microsoft Excel spreadsheet and confirmed by Statistica 8.0 (StatSoft, Inc., Palo Alto, CA, USA, 2001).

### 3.4. Chemical and Structural Characterization of Chitin and Chitosan

Scanning Electron Microscopy (SEM) images were obtained on a JEOL JSM-6400 microscope (Westmont, IL, USA), with acceleration voltage of up to 40 kV and resolution of 36 Å. The samples were prepared on a metal slide with a double-sided adhesive sticker, and covered with a thin layer of gold.

Crystallinity (DRX) was assessed with a PHILIPS X'PERT MPD automatic diffractometer (Almelo, The Netherlands), equipped with a goniometer PW3050 (θ–2θ), at a generator power of 45 kV and 40 mA. The measurements were made at room temperature with Cu Kα1 radiation (wavelength 1.54056 Å), with a graphite monochromator and confocalised geometry (Bragg-Brentano). The step size (2θ) of the measurements was set at 0.040°, and the duration of the step at 1 s. The angular range studied was 5° to 40° 2θ. The percentage of crystalline material in chitin is expressed by a crystallinity index (ICr), determined according to the method proposed by Segal [36].

The deacetylation degree (*DD*) was determined by Proton Nuclear Magnetic Resonance (^1^H-NMR) performed at room temperature on a Bruker Avance II spectrometer (Billerica, MA, USA) at a resonance frequency of 400 MHz. Mestrenova 10.0 software (Mestrelab Research, Santiago de Compostela, Spain) was used for spectral processing. Chitosan samples were dissolved in 0.056 M deuterated trifluoroacetic acid (TFA-d in D_2_O) at a concentration of 7 g/L.

The molecular weight (Mw) was measured by gel permeation chromatography (GPC). The chromatographic system consists of a Waters 625 LC System pump connected to an Ultrahydrogel column (300 × 7.8 mm, Waters, Milford, MA, USA), thermostated in an oven at 35 °C. The detectors were refractive index (Waters 2414), coupled in line with Evaporative Light Scattering (ELS Waters 2424). The mobile phase was 0.2M CH_3_COOH/0.15M CH_3_COONH_4_ working under a flow rate of 0.5 mL/min and an injection volume of 20 μL. The chitosan samples were prepared at a concentration of 1 mg/mL in the same mobile phase solution. All of the solvents and solutions were filtered through 0.45 μm filters (Millipore, Billerica, MA, USA). The standard samples of chitosan were used to establish the molecular weight average of the analyzed samples.

## 4. Conclusions

The pilot plant production of chitin from *P. vannamei* by-products (cephalotorax shells) by a combination of enzymatic and chemical processes was performed. The characteristics of the chitin obtained were: 96% of acetylation degree; 88% of crystallinity; and a very low content of ashes, lipids, proteins, and heavy metals (trace in some cases). Using the response surface methodology, the joint effect of time and NaOH concentration were also studied. The optimal conditions of the deacetylation reaction were obtained at 53.8% of NaOH and 17.5 h of processing in order to produce chitosan with a *DD* of 92% and Mw of 82 kDa. This methodology and the predictive polynomial equation developed can be used for the production of chitosan with defined *DD* values. Further studies performing RSM should be done to produce chitosan from *P. vannamei* with tailored molecular weights.

## Figures and Tables

**Table 1:** Proximate composition of *P. vanamei* by-products and chitin extracted.

Proximate Composition		Content
Raw material	Moisture	76.0 ± 0.06% dry base
	Ash	4.76 ± 0.80% dry base
	Protein	55.0 ± 0.50% dry base
	Lipids	1.6 ± 0.2% dry base
Chitin	Ash	1.44 ± 0.07% dry base
	Nitrogen	6.49 ± 0.03% dry base
	Lipids	0.16 ± 0.03% dry base
	C/N	6.69
	DA	96.0% dry base
	Zn	21.36 ppm
	Na	883.2 ppm
	K	132.9 ppm
	Mn	80.72 ppm
	Mg	192.3 ppm
	P	1023.3 ppm
	Ca	2892 ppm
	Sc	21.3 ppm
	Se	0.24 ppm
	Cu	2.37 ppm
	Fe	33.24 ppm
	Hg	0.12 ppm
	Cd	0.31 ppm
	Pb	1.13 ppm

DA: is the degree of acetylation; C/N: is the ratio carbon/nitrogen.

**Table 2:** Results of the rotatable second-order design of the combined effect of time (*t*) and alkali concentration (*NaOH*) on the production of chitosan (as degree of deacetylation) according to Equation (3) and statistical analysis of significance of the proposed model. The natural values of experimental conditions and the corresponding units are in brackets.

***t***	**NaOH**		***DD* (%)**	***DD_p_* (%)**	**Coefficients**	***t*-Student**	**Equation**
−1 (4.4 h)	−1 (35.9%)		54.0	44.0	87.37	50.79	**87.37**
1 (20.6 h)	−1 (35.9%)		19.0	28.9	12.69	9.32	**12.69 *t***
−1 (4.4 h)	1 (64.1%)		33.0	12.6	4.54	3.33	**4.54 *NaOH***
1 (20.6 h)	1 (64.1%)		79.0	78.5	20.25	10.53	**20.25 *t NaOH***
−1.41 (1 h)	0 (50%)		21.0	40.4	−14.64	9.99	**−14.64 *t^2^***
1.41 (24 h)	0 (50%)		85.0	76.2	−31.74	21.67	**−31.74 *NaOH^2^***
0 (12.5 h)	−1.41 (30%)		20.0	17.9	-	-	-
0 (12.5 h)	1.41 (70%)		18.0	30.7	-	-	-
0 (12.5 h)	0 (50%)		92.0	87.4	-	-	-
0 (12.5 h)	0 (50%)		83.0	87.4	Average value	58.92	-
0 (12.5 h)	0 (50%)		84.0	87.4	Expected average value	87.40	-
0 (12.5 h)	0 (50%)		88.0	87.4	Var (E_e_)	14.8	-
0 (12.5 h)	0 (50%)		90.0	87.4	*t* (α < 0.05; ν = 4)	2.776	-
	**SS**	**ν**	**ν**	**QM**	**Mean Square Ratios**	**F-Fisher tests**	
**Model (M)**	10,822.8	-	5	2164.6	QM_M_/QME = 11.73	$F_{7}^{5}\left(\alpha =0.05\right)=3.972$	
**Error (E)**	1292.2	-	7	184.6	QM(_M+LF_)/QM_M_ = 0.696	$F_{5}^{8}\left(\alpha =0.05\right)=4.818$	
**Exp. Error (E_e_)**	59.2	4	-	14.8	QM_E_/QM_Ee_ = 12.47	$F_{4}^{7}\left(\alpha =0.05\right)=6.094$	
**Lack of Fit (LF)**	1233.0	3	-	411.0	QM_LF_/QM_Ee_ = 27.77	$F_{4}^{3}\left(\alpha =0.05\right)=6.591$	
**Total**	12,114.9		12	-	*R*^2^ = 0.893	-	-
-	-	-	-	-	$R_{adj}^{2}$ = 0.817	-	-

*DD*: experimental degree of deacetylation; *DD_p_*: predicted degree of deacetylation; NS: non-significant coefficient; SS: sum of squares; ν: degrees of freedom; QM: quadratic means of model (M), total error (E), experimental error (E_e_) and lack of fit (LF). Independent variables according to Table 3.

**Table 3:** Experimental domain and codification of independent variables in the factorial rotatable design for chitin deacetylation.

Coded Values	Natural Values	
	NaOH (%)	*t* (h)
−1.41	30	1
−1	35.9	4.4
0	50	12.5
+1	64.1	20.6
+1.41	70	24
Codification: *V_c_* = (*V_n_* − *V*_0_)/Δ*V_n_*		
Decodification: *V_n_* = *V*_0_ + (Δ*V_n_* × *V_c_)*		
*V*_0_ = natural value in the centre of the domain		
*V_n_* = natural value of the variable to codify		
*V_c_* = codified value of the variable		
Δ*V_n_* = increment of *V_n_* for unit of *V_c_*		

## References

[1] Food and Agriculture (FAO) (2016). The State of World Fisheries and Aquaculture. Contributing to Food Security and Nutrition for All.

[2] Kumar M.N.R. (2000). A review of chitin and chitosan applications. React. Funct. Polym..

[3] Jayakumar R., Prabaharan M., Kumar P.S., Nair S., Tamura H. (2011). Biomaterials based on chitin and chitosan in wound dressing applications. Biotechnol. Adv..

[4] Ferhat M., Kadouche S., Drouiche N., Messaoudi K., Messaoudi B., Lounici H. (2016). Competitive adsorption of toxic metals on bentonite and use of chitosan as flocculent coagulant to speed up the settling of generated clay suspensions. Chemosphere.

[5] Bouhenna M., Salah R., Bakour R., Drouiche N., Abdi N., Grib H., Lounici H., Mameri N. (2015). Effects of chitin and its derivatives on human cancer cells lines. Environ. Sci. Pollut. Res..

[6] Shahidi F., Arachchi J.K.V., Jeon Y.-J. (1999). Food applications of chitin and chitosans. Trends Food Sci. Technol..

[7] Jeon Y.-J., Shahidi F., Kim S.-K. (2000). Preparation of chitin and chitosan oligomers and their applications in physiological functional foods. Food Rev. Int..

[8] Tharanathan R.N., Kittur F.S. (2003). Chitin—The undisputed biomolecule of great potential. Crit. Rev. Food Sci. Nutr..

[9] Tolaimate A., Desbrieres J., Rhazi M., Alagui A. (2003). Contribution to the preparation of chitins and chitosans with controlled physico-chemical properties. Polymer.

[10] Synowiecki J., Al-Khateeb N.A. (2003). Production, properties, and some new applications of chitin and its derivatives. Crit. Rev. Food Sci. Nutr..

[11] Benhabiles M., Salah R., Lounici H., Drouiche N., Goosen M., Mameri N. (2012). Antibacterial activity of chitin, chitosan and its oligomers prepared from shrimp shell waste. Food Hydrocoll..

[12] Vázquez J.A., Caprioni R., Nogueira M., Menduiña A., Ramos P., Pérez-Martín R.I. (2016). Valorisation of effluents obtained from chemical and enzymatic chitin production of *Illex argentinu*s pen by-products as nutrient supplements for various bacterial fermentations. Biochem. Eng. J..

[13] Benhabiles M.S., Abdi N., Drouiche N., Lounici H., Pauss A., Goosen M.F.A., Mameri N. (2013). Protein recovery by ultrafiltration during isolation of chitin from shrimp shells *Parapenaeus longirostris*. Food Hydrocoll..

[14] Gildberg A., Stenberg E. (2001). A new process for advanced utilisation of shrimp waste. Process Biochem..

[15] Arancibia M.Y., Alemán A., Calvo M.M., López-Caballero M.E., Montero P., Gómez-Guillén M.C. (2014). Antimicrobial and antioxidant chitosan solutions enriched with active shrimp (*Litopenaeus vannamei*) waste materials. Food Hydrocoll..

[16] Kurita K., Akao H., Yang J., Shimojoh M. (2003). Nonnatural branched polysaccharides: Synthesis and properties of chitin and chitosan having disaccharide maltose branches. Biomacromolecules.

[17] Younes I., Ghorbel-Bellaaj O., Nasri R., Chaabouni M., Rinaudo M., Nasri M. (2012). Chitin and chitosan preparation from shrimp shells using optimized enzymatic deproteinization. Process Biochem..

[18] Vázquez J.A., Rodríguez-Amado I., Montemayor M.I., Fraguas J., González M.d.P., Murado M.A. (2013). Chondroitin sulfate, hyaluronic acid and chitin/chitosan production using marine waste sources: Characteristics, applications and eco-friendly processes: A review. Mar. Drugs.

[19] Majekodunmi S.O. (2016). Current development of extraction, characterization and evaluation of properties of chitosan and its use in medicine and pharmaceutical industry. Am. J. Polym. Sci..

[20] Duan S., Li L., Zhuang Z., Wu W., Hong S., Zhou J. (2012). Improved production of chitin from shrimp waste by fermentation with epiphytic lactic acid bacteria. Carbohydr. Polym..

[21] Jung W., Jo G., Kuk J., Kim Y., Oh K., Park R. (2007). Production of chitin from red crab shell waste by successive fermentation with *Lactobacillus paracasei* kctc-3074 and serratia marcescens fs-3. Carbohydr. Polym..

[22] Younes I., Ghorbel-Bellaaj O., Chaabouni M., Rinaudo M., Souard F., Vanhaverbeke C., Jellouli K., Nasri M. (2014). Use of a fractional factorial design to study the effects of experimental factors on the chitin deacetylation. Int. J. Biol. Macromol..

[23] Younes I., Hajji S., Rinaudo M., Chaabouni M., Jellouli K., Nasri M. (2016). Optimization of proteins and minerals removal from shrimp shells to produce highly acetylated chitin. Int. J. Biol. Macromol..

[24] Jiang Q.-H., Ye S.-Q., Wang Y. (2013). Optimization of the preparation of chitosan from wastes of *Penaeus vannamei*. Mod. Food Sci. Technol..

[25] Demim S., Drouiche N., Aouabed A., Benayad T., Couderchet M., Semsari S. (2014). Study of heavy metal removal from heavy metal mixture using the CCD method. J. Ind. Eng. Chem..

[26] Demim S., Drouiche N., Aouabed A., Benayad T., Dendene-Badachee O., Semsari S. (2013). Cadmium and nickel:Assessment of the physiological effects and heavy metal removal using a response surface approach by *L. gibba*. Ecol. Eng..

[27] Blanco M., Fraguas J., Sotelo C.G., Pérez-Martín R.I., Vázquez J.A. (2015). Production of chondroitin sulphate from head, skeleton and fins of *Scyliorhinus canicula* by-products by combination of enzymatic, chemical precipitation and ultrafiltration methodologies. Mar. Drugs.

[28] Gopalakannan A., Jasmine G.I., Shanmugam S., Sugumar G. (2000). Application of proteolytic enzyme, papain for the production of chitin and chitosan from shrimp waste. J. Mar. Biol. Assoc. India.

[29] Allwin S.I.J., Jeyasanta K.I., Patterson J. (2015). Extraction of chitosan from white shrimp (*Litopenaeus vannamei*) processing waste and examination of its bioactive potentials. Adv. Biol. Res..

[30] Gagne N., Simpson B. (1993). Use of proteolytic enzymes to facilitate the recovery of chitin from shrimp wastes. Food Biotechnol..

[31] Gartner C., Peláez C.A., López B.L. (2010). Characterization of chitin and chitosan extracted from shrimp shells by two methods. e-Polymers.

[32] Dziril M., Grib H., Laribi-Habchi H., Drouiche N., Abdi N., Lounici H., Pauss A., Mameri N. (2015). Chitin oligomers and monomers production by coupling γ radiation and enzymatic hydrolysis. J. Ind. Eng. Chem..

[33] Xu J., McCarthy S.P., Gross R.A., Kaplan D.L. (1996). Chitosan film acylation and effects on biodegradability. Macromolecules.

[34] Kasaai M. (2007). A review of several reported procedures to determinate the degree of *N*-acetylation for chitin and chitosan using infrared spectroscopy. Carbohydr. Polym..

[35] Cárdenas G., Cabrera G., Taboada E., Miranda S.P. (2004). Chitin characterization by SEM, FTIR, XRD, and 13C cross polarization/mass angle spinning NMR. J. Appl. Polym. Sci..

[36] Segal L., Creely J., Martin A., Conrad C. (1959). An empirical method for estimating the degree of crystallinity of native cellulose using the x-ray diffractometer. Text. Res. J..

[37] Lopes C., Antelo L.T., Franco-Uría A., Alonso A.A., Pérez-Martín R. (2017). Chitin production from crustacean biomass: Sustainability assessment of chemical and enzymatic processes. J. Clean. Prod..

[38] Commission Regulation (ec) no 629/2008 of 2 July 2008 Amending Regulation (ec) no 1881/2006 Setting Maximum Levels for Certain Contaminants in Foodstuffs. http://eur-lex.europa.eu/legal-content/EN/ALL/?uri=CELEX%3A32008R0629.

[39] Cudennec B., Ravallec-Plé R., Courois E., Fouchereau-Peron M. (2008). Peptides from fish and crustacean by-products hydrolysates stimulate cholecystokinin release in stc-1 cells. Food Chem..

[40] Goosen N., de Wet L., Görgens J. (2015). Comparison of hydrolysed proteins from different raw materials in diets for mozambique tilapia *Oreochromis mossambicus*. Aquac. Int..

[41] Benhabiles M., Abdi N., Drouiche N., Lounici H., Pauss A., Goosen M., Mameri N. (2012). Fish protein hydrolysate production from sardine solid waste by crude pepsin enzymatic hydrolysis in a bioreactor coupled to an ultrafiltration unit. Mater. Sci. Eng. C.

[42] Amado I.R., González M., Murado M.A., Vázquez J.A. (2016). Shrimp wastewater as a source of astaxanthin and bioactive peptides. J. Chem. Technol. Biotechnol..

[43] Amado I.R., Vázquez J.A., Murado M.A., González M.P. (2015). Recovery of astaxanthin from shrimp cooking wastewater: Optimization of astaxanthin extraction by response surface methodology and kinetic studies. Food Bioprocess Technol..

[44] García-López M., Pérez-Martín R.I., Sotelo C.G. (2016). Carotenoid pigments composition of two commonly discarded decapod crustaceans in grand sole and the Galician-Northern Portugal coast fisheries. J. Aquat. Food Prod. Technol..

[45] Yoon G.A., Kim Y.M., Chi G.Y., Hwang H.J. (2005). Effects of tuna bone and herbal extract on bone metabolism in ovariectomized rats. Nutr. Res..

[46] Herpandi N.H., Rosma A., Wan Nadiah W. (2011). The tuna fishing industry: A new outlook on fish protein hydrolysates. Compr. Rev. Food Sci. Food Saf..

[47] Zhao Y., Park R.-D., Muzzarelli R.A. (2010). Chitin deacetylases: Properties and applications. Mar. Drugs.

[48] Raymond L., Morin F.G., Marchessault R.H. (1993). Degree of deacetylation of chitosan using conductometric titration and solid-state nmr. Carbohydr. Res..

[49] Paulino A.T., Simionato J.I., Garcia J.C., Nozaki J. (2006). Characterization of chitosan and chitin produced from silkworm crysalides. Carbohydr. Polym..

[50] Ottøy M.H., Vårum K.M., Christensen B.E., Anthonsen M.W., Smidsrød O. (1996). Preparative and analytical size-exclusion chromatography of chitosans. Carbohydr. Polym..

[51] Wardhani D.H., Vázquez J.A., Pandiella S.S. (2010). Optimisation of antioxidants extraction from soybeans fermented by *Aspergillus oryzae*. Food Chem..

[52] Weska R., Moura J.M.D., Batista L.D.M., Rizzi J., Pinto L.A.D.A. (2007). Optimization of deacetylation in the production of chitosan from shrimp wastes: Use of response surface methodology. J. Food Eng..

[53] Association of Official Analytical Chemists (AOAC) (1990). Methods of Analysis.

[54] Bligh E.G., Dyer W.J. (1959). A rapid method of total lipid extraction and purification. Can. J. Biochem. Phys..

[55] Box G.E., Hunter J.S., Hunter W.G. (2005). Statistics for Experimenters: Design, Innovation, and Discovery.

